# Supplementary material for: Mapping the Genetic Architecture of the Adaptive Integrated Stress Response in S. cerevisiae
Source: bioRxiv. 2024 Dec 22:2024.12.19.629525. Preprint. [Version 1] doi: 10.1101/2024.12.19.629525 (PMC11702766; doi:10.1101/2024.12.19.629525)
Supplement: Supplement 1 [file media-1.pdf]

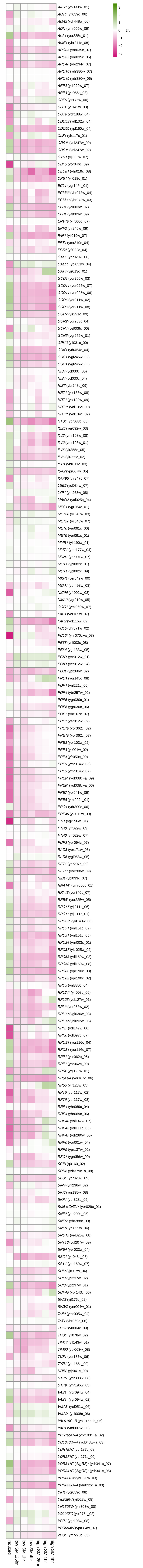

Supplementary File 1: Heatmap of all single mutants shows ISR log2 fold-change normalized to negative guide reference. Guides marked with a * reflect updated guide targets at divergent promoters that were misassigned in the original table.
