# Supplementary material for: Mapping the Genetic Architecture of the Adaptive Integrated Stress Response in S. cerevisiae
Source: bioRxiv. 2024 Dec 22:2024.12.19.629525. Preprint. [Version 1] doi: 10.1101/2024.12.19.629525 (PMC11702766; doi:10.1101/2024.12.19.629525)
Supplement: Supplement 5 [file NIHPP2024.12.19.629525v1-supplement-5.pdf]

bioRxiv preprint doi: https://doi.org/10.1101/2024.12.19.629525; this version posted December 22, 2024. The copyright holder for this preprint (which was not certified by peer review) is the author/funder, who has granted bioRxiv a license to display the preprint in perpetuity. It is made available under aCC-BY 4.0 International license.

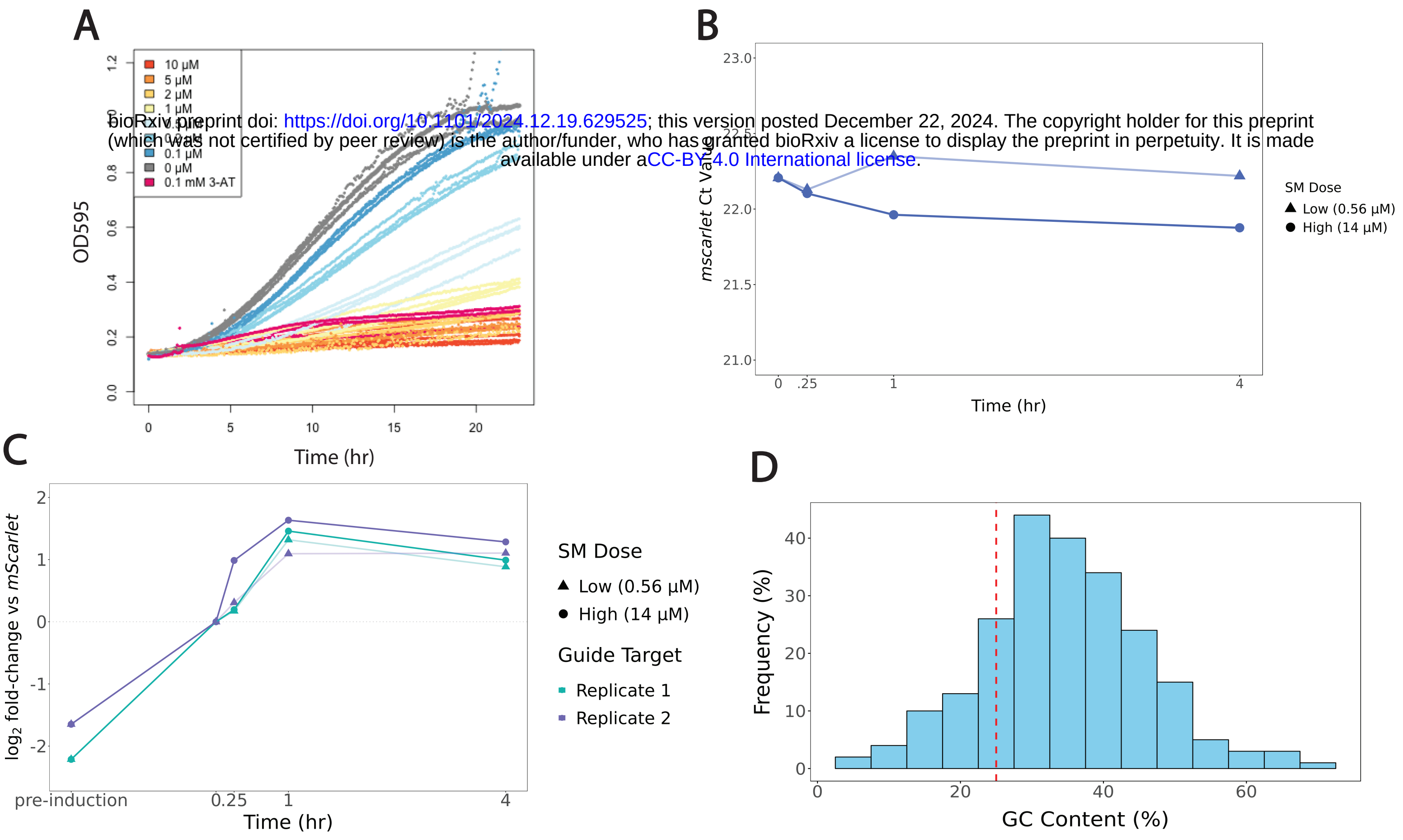

Supplementary Figure 1:

Additional pilot data

(A) Raw data growth curves from Figure 4.1A **(B)** RT-qPCR for *UBC6* driven mScarlet normalizer reporter shows minimal variability in Ct values for low SM dose (●) or high SM dose (▲) treated cells after 0, .25, 1, or 4 hours. **(C)** RT-qPCR of ISR reporter normalized to mScarlet for CiBER-seq libraries. Two biological replicates for low and high dose SM treatment. **(D)** Histogram of percentage of GC content in 224 CiBER-seq spacer sequences. Dashed line represents 25% cutoff mark; spacer sequences that fell below this threshold were not followed-up on due to increased likelihood of off-target effects (Konstantakos et al. 2022).

bioRxiv preprint doi: https://doi.org/10.1101/2024.12.19.629525; this version posted December 22, 2024. The copyright holder for this preprint (which was not certified by peer review) is the author/funder, who has granted bioRxiv a license to display the preprint in perpetuity. It is made available under aCC-BY 4.0 International license.

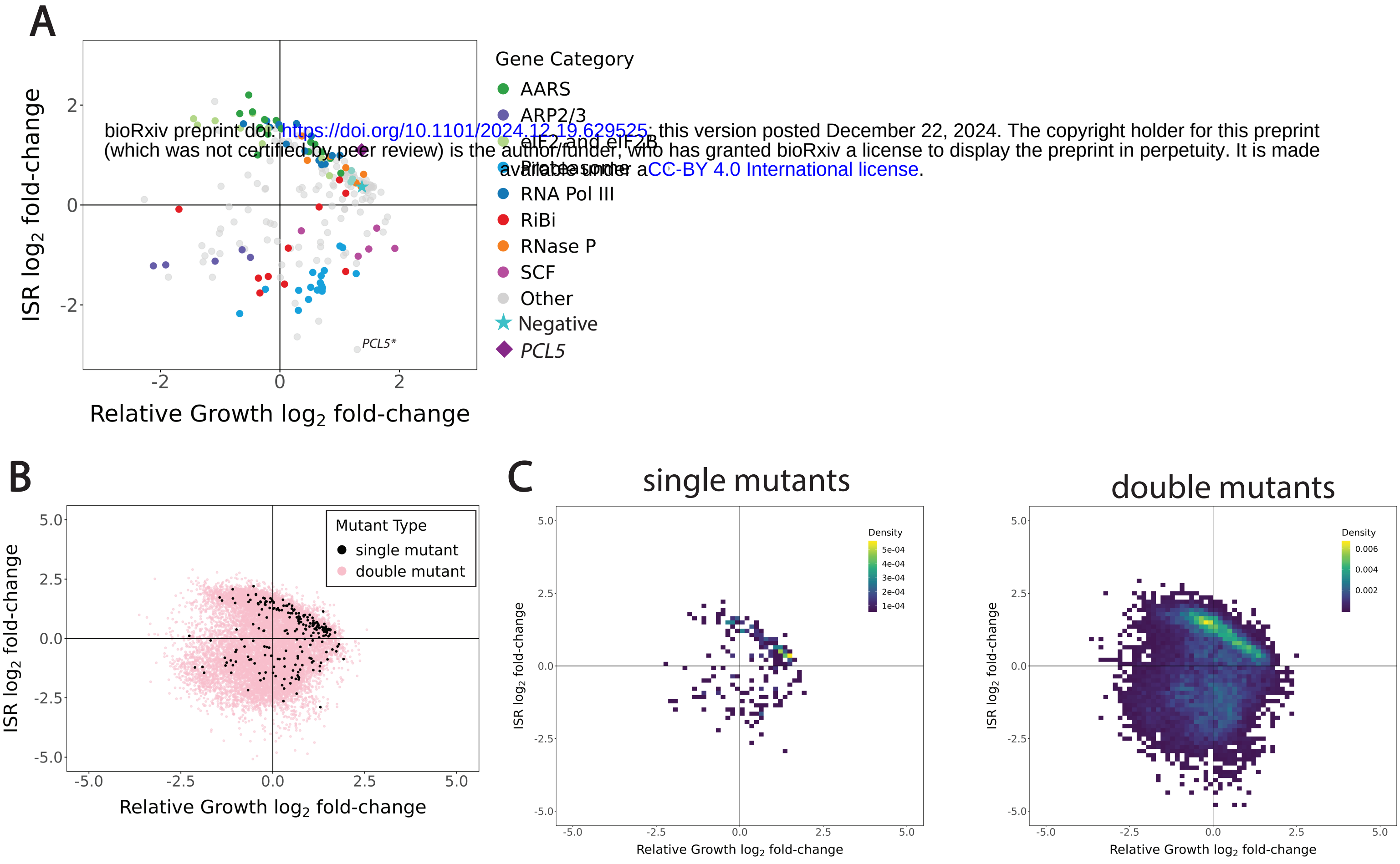

Supplementary Figure 2:

Categorized single mutant analysis and double mutant analysis
(A) Guides targeting functionally related genes show similar growth and ISR activation phenotypes after guide-induction. **(B)** Dual-guide phenotypes (pink) and single-guide phenotypes (black) from DESeq2 analysis **(C)** Single-guide (left) and double-guide density plots (right) show similar density patterns within the range of the single-mutant phenotypes. Many guides activate the ISR and cause cellular fitness defects in a proportionate way.

bioRxiv preprint doi: https://doi.org/10.1101/2024.12.19.629525; this version posted December 22, 2024. The copyright holder for this preprint (which was not certified by peer review) is the author/funder, who has granted bioRxiv a license to display the preprint in perpetuity. It is made available under aCC-BY 4.0 International license.

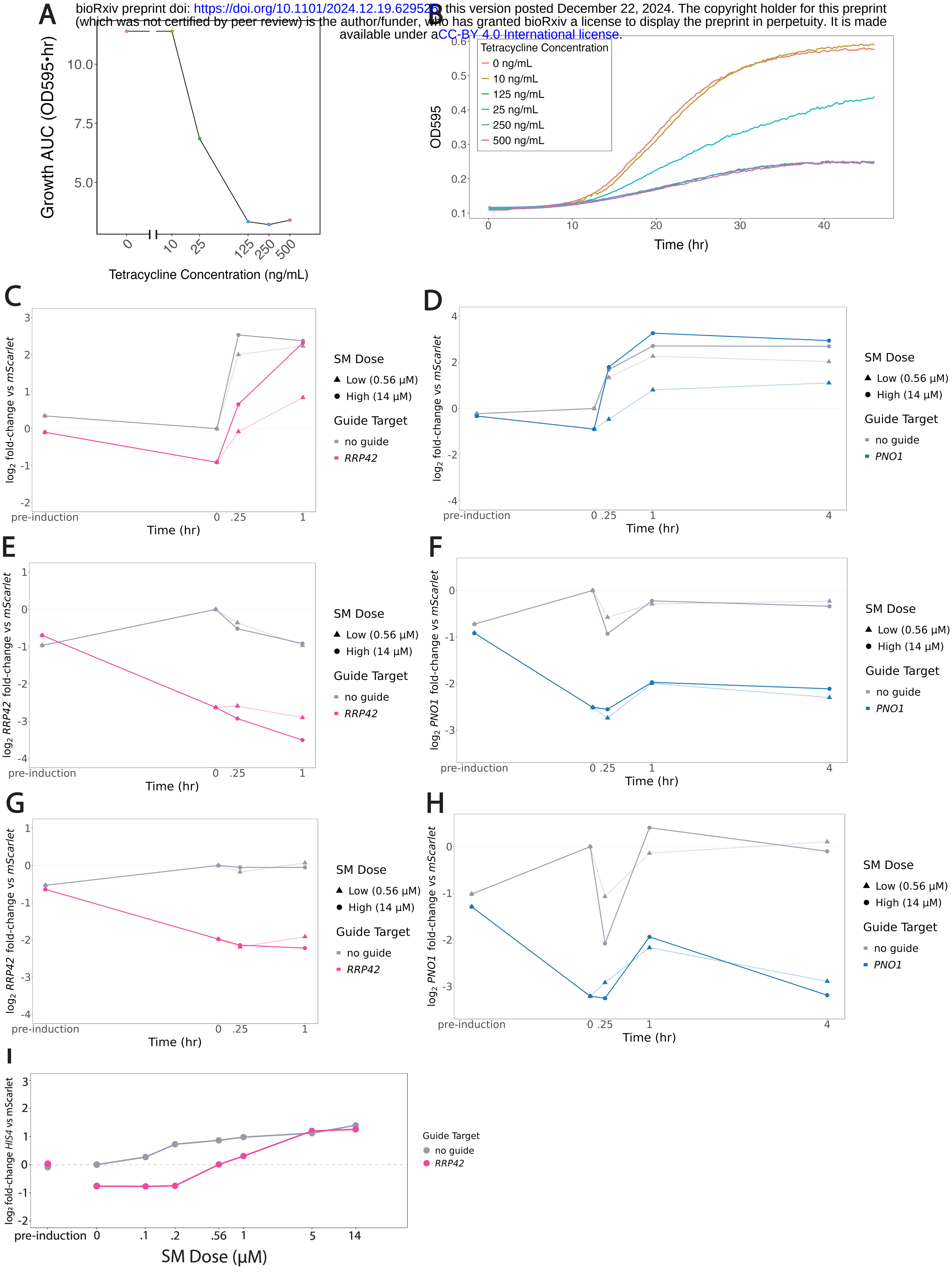

bioRxiv preprint doi: https://doi.org/10.1101/2024.12.19.629525; this version posted December 22, 2024. The copyright holder for this preprint (which was not certified by peer review) is the author/funder, who has granted bioRxiv a license to display the preprint in perpetuity. It is made available under aCC-BY 4.0 International license.

Supplementary Figure 3:

Replicates of *RRP42* and *PNO1* lead to SM treatment dose-dependent response

(A) Growth AUC (Area Under Logistic Curve) reflects relative growth rate for fitted growth curves for tetracycline guide-induction dose response. Growth rate slows with increased knockdown efficiency. **(B)** Raw growth curves from (A). **(C)** RT-qPCR of ISR reporter for wildtype and *RRP42* knockdown cells, replicate 2 of Figure 3C. **(D)** RT-qPCR of ISR reporter for wildtype and *PNO1* knockdown cells, replicate 2 of Figure 3D. **(E)** RT-qPCR *RRP42* knockdown-efficiency of data from Figure 3C **(F)** RT-qPCR *PNO1* knockdown-efficiency of Figure 3D. **(G)** RT-qPCR *RRP42* knockdown-efficiency of data from Supp Figure 3C. **(H)** RT-qPCR *PNO1* knockdown-efficiency of data from Supp Figure 3D. **(I)** RT-qPCR for endogenous *HIS4* target for SM dose response from Figure 3E. HIS4 expression in *RRP42* knockdown cells shows similar decrease to ISR reporter.

bioRxiv preprint doi: https://doi.org/10.1101/2024.12.19.629525; this version posted December 22, 2024. The copyright holder for this preprint (which was not certified by peer review) is the author/funder, who has granted bioRxiv a license to display the preprint in perpetuity. It is made available under aCC-BY 4.0 International license.

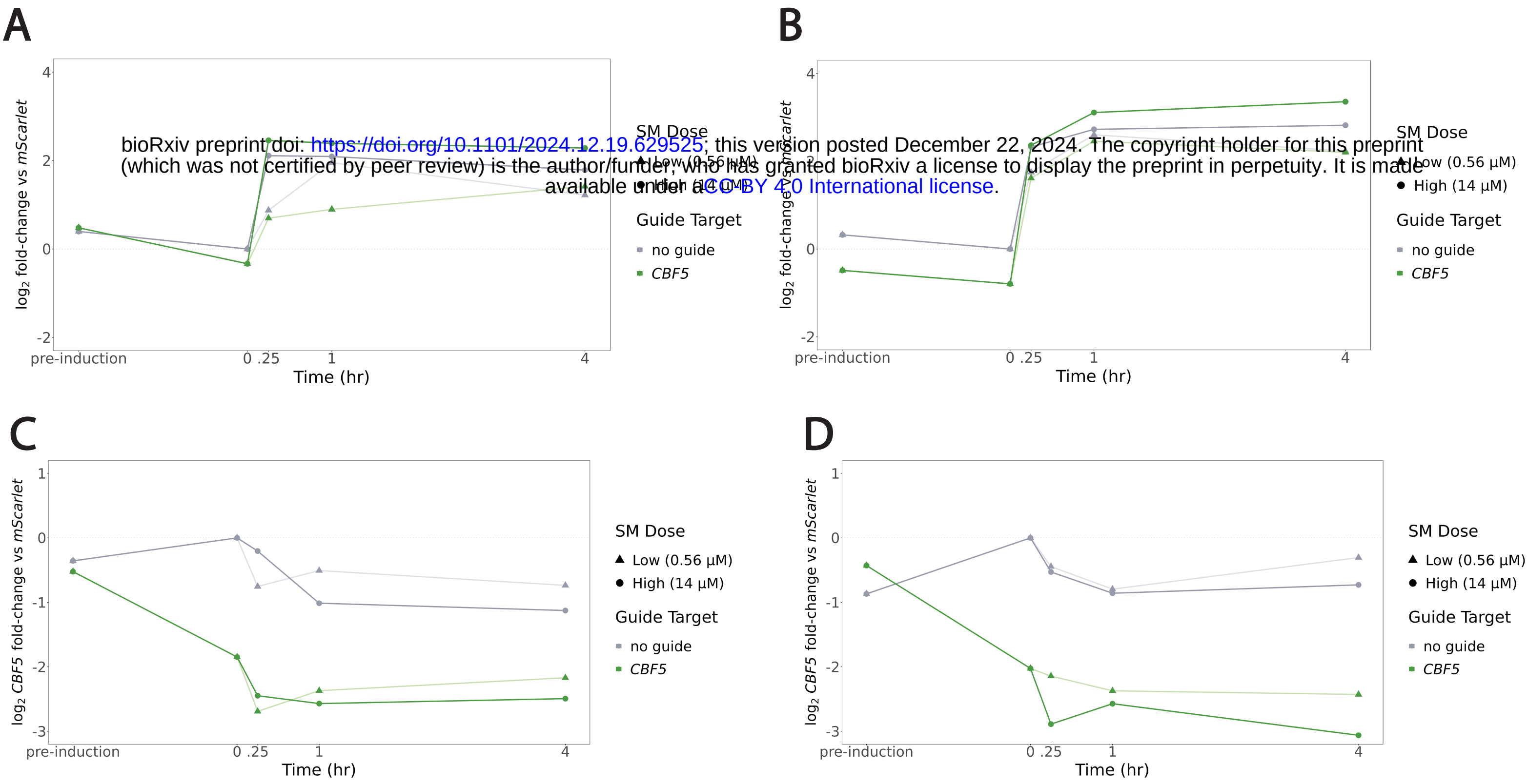

Supplementary Figure 4:

Knockdown of RiBi gene *CBF5* SM dose-dependent effect
(A) RT-qPCR of ISR reporter for wildtype and *PNO1* knockdown cells, replicate 1. ISR reporter normalized to mScarlet for pre-guide induction, post-guide induction and 0, .25, 1 and 4 hour low or high SM treated cells. Low SM dose reduces ISR activation and high SM dose strengthens ISR activation. **(B)** RT-qPCR of ISR reporter for wildtype and *CBF5* knockdown cells, replicate 2 of Supp Figure 4B. **(C)** RT-qPCR *CBF5* knockdown-efficiency of data from Supp Figure 4A. **(D)** RT-qPCR *CBF5* knockdown-efficiency of data from Supp Figure 4B.

bioRxiv preprint doi: https://doi.org/10.1101/2024.12.19.629525; this version posted December 22, 2024. The copyright holder for this preprint (which was not certified by peer review) is the author/funder, who has granted bioRxiv a license to display the preprint in perpetuity. It is made available under aCC-BY 4.0 International license.

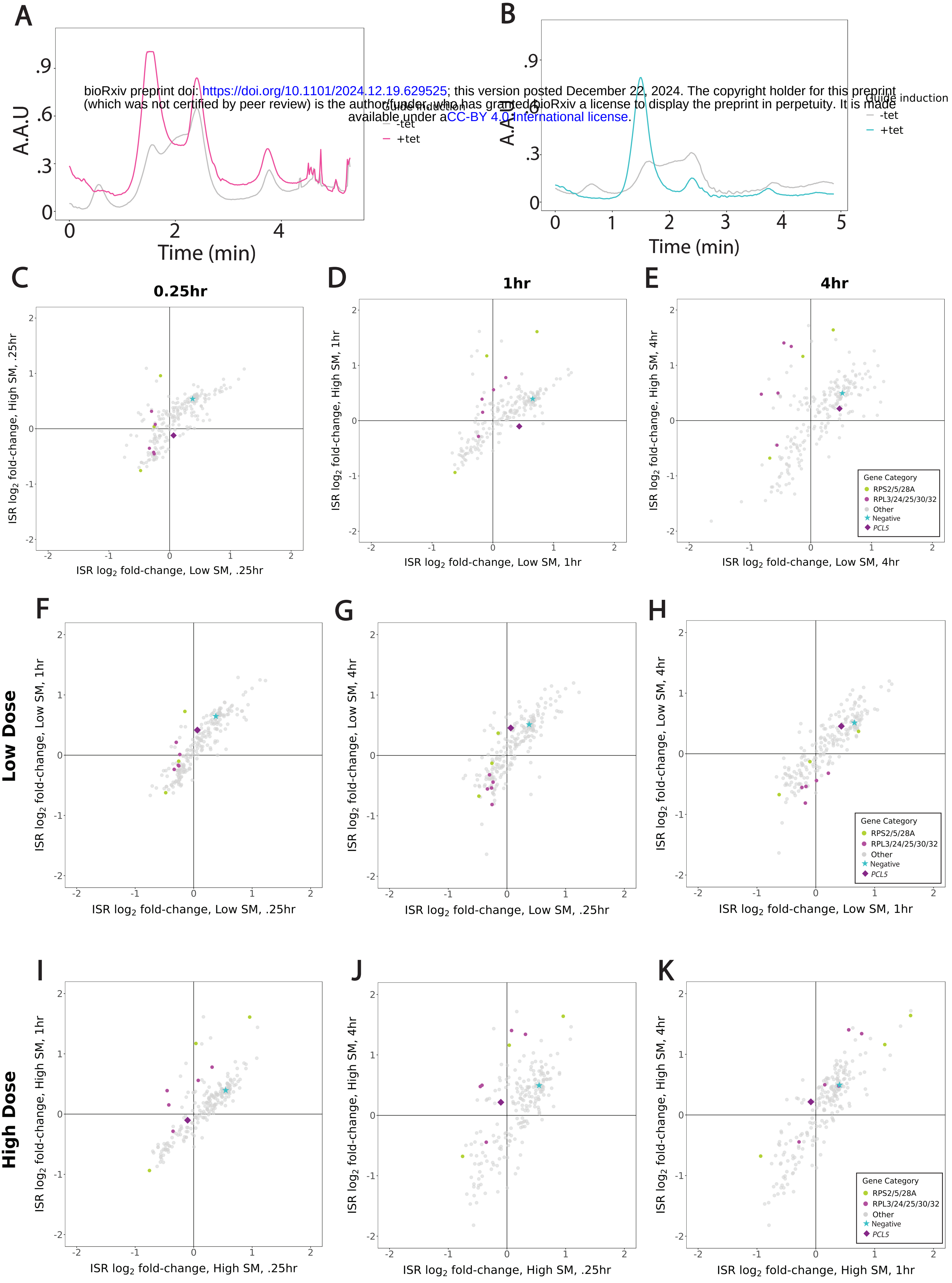

bioRxiv preprint doi: https://doi.org/10.1101/2024.12.19.629525; this version posted December 22, 2024. The copyright holder for this preprint (which was not certified by peer review) is the author/funder, who has granted bioRxiv a license to display the preprint in perpetuity. It is made available under aCC-BY 4.0 International license.

Supplementary Figure 5:

Targeting ribosomal genes effects ISR dynamics
(A-B) Short 5-30% sucrose gradients for **(A)** *RRP42* knockdown and **(B)** *PNO1* knockdown lysates. **(C-E)** Scatterplots highlighting guides targeting small and large ribosome subunits for low versus high SM treatment dose for **(C)** .25 hour **(D)** 1 hour **(E)** 4 hours. (F-H) Scatterplots highlighting guides targeting small and large ribosome subunits for low dose SM treatment **(F)** .25hr versus 1hr timepoints **(G)** .25hr versus 4hr timepoints **(H)** 1hr versus 4 hour timepoints. **(I-K)** Scatterplots highlighting guides targeting small and large ribosome subunits for high dose SM treatment **(I)** .25hr versus 1hr timepoints **(J)** .25hr versus 4hr timepoints **(K)** 1hr versus 4 hour timepoints.
